# Preproject ‘Swiss Virtual Natural History Collection’

**DOI:** 10.1093/database/baad072

**Published:** 2023-10-31

**Authors:** Ana Petrus, Tobias Wildi, Stefanie Müller

**Affiliations:** Swiss Institute for Information Science (SII), University of Applied Sciences of the Grisons, Ringstrasse 34, Chur 7000, Switzerland; Swiss Institute for Information Science (SII), University of Applied Sciences of the Grisons, Ringstrasse 34, Chur 7000, Switzerland; Swiss Institute for Information Science (SII), University of Applied Sciences of the Grisons, Ringstrasse 34, Chur 7000, Switzerland

## Abstract

The initiative ‘Swiss Natural History Collection Network’ (SwissCollNet) aims to improve the accessibility of the Swiss natural history collections for research, education and the wider public. In the 2021–24 funding period, SwissCollNet is identifying, prioritizing and digitizing as many collections as possible and building an online portal called the ‘Swiss Virtual Natural History Collection’ (hereafter SVNHC) to provide open access to harmonized sample data from Swiss collections. The University of Applied Sciences of the Grisons developed scenarios for the technical implementation of the SVNHC portal in a preliminary study on behalf of SwissCollNet and based on the requirements of collections and data centres.

## Introduction

The ‘Swiss Natural History Collection Network’ (SwissCollNet) is an initiative committed to improving the accessibility of natural history collections for research, education and society (https://swisscollnet.scnat.ch/de). During the funding period 2021–24, SwissCollNet is identifying, prioritizing and digitizing as many collections as possible and building an online portal, the Swiss Virtual Natural History Collection (hereafter SVNHC), which will provide open access to harmonized specimen data from the Swiss collections (https://swisscollnet.scnat.ch/de/application) for researchers, data providers, curators, collection-holding institutions and customers from the cantons and the federal administration in Switzerland, as well as citizen scientists. As a result, it is hoped the collections will gain increased public visibility.

In order to identify the most suitable and unbiased solution to increase digital access to collection data currently decentrally stored in numerous public collection-holding institutions, SwissCollNet has mandated the authors of this paper to conduct a preparatory study, the Preproject ‘Swiss Virtual Natural History Collection’ (SVNHC) (1). We started this study as outsiders without previous exposure to biological or geological collections.

This document presents a summary of the methodology, results and recommendations of the study.

## Materials and methods

As a starting point, we identified which SwissCollNet goals justify the harmonization and aggregation of collection data stored in Swiss collection-holding institutions and formulated prerequisites to reach these goals.

Second, we interviewed 22 people either working in collection-holding institutions (curators and IT-system administrators) or representatives of national and international data aggregators. The interviews were conducted based on an expert questionnaire containing the same questions for all interviewees covering five main topics. The representatives were asked about (i) solutions their institutions used to manage and transmit collection data, their institutions’ involvement in sharing data internationally, open access and FAIR principles, as well as urgent problems and needs; (ii) their future visions to increase accessibility to collection data and about current national and international initiatives, organizations and infrastructure dealing with collection data; (iii) what their preferred funding solution looks like after initial federal payment; (iv) whether they use any technical tools to manage metadata in order to make the information interoperable for different IT solutions; and (v) how adapted they are to connect national and international solutions, i.e. use of application programming interfaces (APIs) to connect databases and repositories (for further information, see Table 1).

**Table 1.** Detailed information on the questionnaire used to interview representatives of Swiss museums and collections

Main topic	Question	Intention
Solution approach	What do you use today for collection management?	Evaluation of the currently used CMSs and tools by selected Swiss collections and museums.
Solution approach	Are you involved in international communities?	Evaluation of the current international networking and connection of various institutions.
Solution approach	What does your data look like?	Gain an impression of data characteristics such as structure and scope.
Solution approach	Do you have an open data policy?	Gain an impression of how the data is handled in terms of accessibility, reusability, legal issues, etc.
Solution approach	Where do you see problems with the current solutions your institution is working with?	Get a picture of how the institution describes itself and where they think are the biggest challenges to face.
Solution approach	What are your most urgent needs with regard to data management?	Get a picture of where the most urgent changes should be made regarding data management.
Vision	How would an ideal solution for aggregating different collections have to be designed (national/international, centralized/decentralized, develop it yourself/standard product and commercial licence okay/exclusive open source)?	Get an impression of how the institutions envision an ideal solution with regard to the listed points. This should lead to different possibilities of how such a solution could be worked out and what requirements should be fulfilled.
Vision	Where (on which infrastructure) could a national aggregation solution be operated?	Get an idea of what the institutions envision where a common solution could be hosted.
Vision	Who could take responsibility for operation (design of operation, support and further development after commissioning)?	Get an idea of what the institutions envision who might be responsible for a joint solution in the long term, and to what extent they see themselves as responsible.
Commercial model	Which commercial model would you prefer? After a federal initial payment, further development of a collection aggregating solution will be funded by (i) ongoing contributions from each collection, (ii) project-oriented contributions or (iii) further support by federal bodies.	Gain an impression of who the institutions see as having financial responsibility so that a common solution can be sustained over the long term.
Management of data/collection	Apart from managing collection data (meaning both contextual metadata, like location and collector of a specimen, and data itself, for example measurements of a specimen and picture of a specimen) do you also use and/or need a solution to manage technical metadata (i.e. properties of a data file) in order to make it easier sharable across different IT solutions?	Gain an impression of the extent to which data are currently being shared or will be shared in the future, and whether it is in the institutions’ interest to share data and collaborate.
Connectivity to national and international solutions	How collaborative on an international level does the collection solution need to be? Are, for example, APIs needed to connect the database to international collections and repositories?	Gain an impression of how international exchange of data is a need for institutions as it would affect infrastructure implementation.
Connectivity to national and international solutions	How would a Swiss-wide natural history collection be embedded in the overall biology research infrastructure as proposed by Biology Roadmap SCNAT https://scnat.ch/en/uuid/i/2b8e86f8-f412-5447-9b78-8a9274733450-Biology_Roadmap? Could it be one module among many?	Gain an impression of whether there is a common vision among the institutions, what a common solution could look like, or whether each institution has its own vision.

Furthermore, we identified existing organizational and technical building blocks that might be enhanced and then used to aggregate collection data. Thereby, building blocks are individual components [e.g. virtual data centre (VDC) and Global Biodiversity Information Facility (GBIF)] that could contribute to an overall infrastructure solution.

In the evaluation of the interviews, similar statements were grouped and thus weighted as more pronounced needs than statements that were given only a few times. In addition, the size of the institution was compared with the statements to examine whether this had an impact on the needs of the institutions. Based on these analyses, requirements for an SVNHC were formulated from the perspective of a variety of stakeholders and a number of scenarios for the realization of an SVNHC were formulated and evaluated.

## Results and discussion

### Goals and prerequisites

Currently, most of the data of the Swiss natural history collections are distributed among a range of institutions and are not fed into centralized portals or aggregators. Access to these dispersed data is difficult not only for scholars but also for the general public as there is no central access point to all collection data in the sense of a national aggregator that acquires, stores and delivers such data for various dissemination purposes. Such an ‘SVNHC’ would greatly increase the rich stock of data stored locally in the collections and have a positive national and international impact. On a national level, the benefits of an SVNHC include facilitated access to collection data for researchers, the Swiss public administration (federal, cantonal and municipal), non-state actors such as ecology and planning offices, engineering company societies, the general public and hobbyists. Furthermore, harmonized data can be fed more easily into international aggregators, internationally increasing the visibility of the Swiss natural history collections and fostering exchange within the international research community.

Prerequisites for building an SVNHC are a sustainable business model and, from a technical point of view, a modular IT architecture that can be easily adapted to future changing demands and developments.

Important aspects for success include support of natural history collections for the digitization of their collections and focus on the harmonization of data standards and digitization processes to achieve homogeneous data. Also, collection-holding institutions must operate and maintain suitable collection management systems (CMSs), which are capable of exporting data in a standardized form and making it available to the aggregator. Between these different levels and actors, interfaces such as metadata standards and protocols for data exchange must be defined. The introduction and improvement of CMSs are infrastructure projects that are ideally carried out jointly by different institutions because this creates synergies in the development and maintenance of software. It must also be ensured that the FAIR data principles (https://www.rd-alliance.org/rda-disciplines/rda-and-biodiversity) are followed and that the SVNHC as a data source is compatible with standards set by research funding agencies such as the Swiss National Science Foundation and European research funding programmes like ‘Horizon Europe’ (https://cinea.ec.europa.eu/programmes/horizon-europe_en). High data quality and reliability are crucial, as is the documentation of the origin of the data (provenance information). Also, an access policy to ensure the data are available in a unified way across all collections participating in SVNHC must be defined, and the data must become as accessible as possible, with access restricted only where legally necessary (e.g. to protect the exact location of endangered species). Interoperability with international data infrastructure like GBIF (https://www.gbif.org/) and possibly Distributed System of Scientific Collections (DiSSCo) (https://www.dissco.eu/) must be guaranteed and data made reusable, while the data sovereignty must be kept at the level of the collections. Furthermore, it is crucial to implement the aggregator on a national level to retain control over financing and further development and avoid becoming dependent on international decision-making processes.

### Summary of interview statements

#### Collection management systems

Currently, the collections and institutions use a large variety of CMSs. The functional and technical requirements of these CMSs, the financial framework of the institutions and the skills of the team members within the institutions vary greatly between the collections and institutions. The CMSs in use are Specify (https://www.specifysoftware.org/), BioOffice (http://www.BioOffice.org/), Digitalis, Botalista (https://botalista.community/), Diversity Workbench, Symbiota (https://symbiota.org/), easyDB (https://www.programmfabrik.de/en/easydb/) and in-house developments based on Oracle, Filemaker or Microsoft Access.

The focus of the collections in terms of content, their budget processes and the functional and technical requirements of the CMS diverge greatly. Collections want to keep their independence in choosing and implementing a CMS, and thus CMS must remain decentralized and under the control of the individual institutions.

#### Digitization of collections

**Figure 1.**
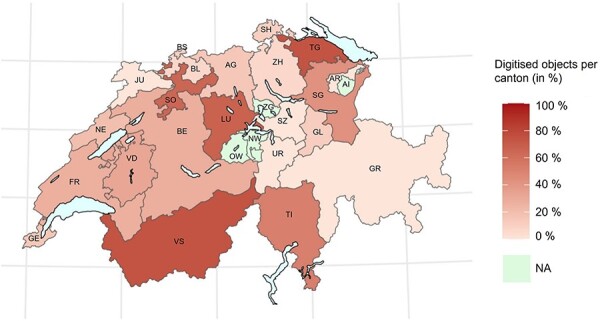
This heat map shows the percentage of digitised objects in natural history collections per canton. There is no available data for four cantons (i.e. Appenzell Innerrhoden, Nidwalden, Obwalden and Zug) (2).

The interviews revealed that only a part of the objects and inventories are digitized (denoting here both the digitization of metadata and visual capture of objects) with the proportion of digitization lower in middle-sized/smaller collections. Beer *et al.* (2) report on the percentage of digitized objects in 56 different museums and collections in 22 cantons. Based on their reported numbers, Figure 1 provides an overview of the digitization progress of objects in Swiss collections and museums in 2018, which are the most current available numbers. The canton of Thurgau has digitized the most objects with 74%, while Zürich and Basel-Landschaft currently have the lowest proportion of their objects digitized with 7%. With these figures, however, it must be noted that there are large differences in the number of objects in the cantons [for more details, see Tables 2 and Beer *et al.* (2)].

**Table 2.** Detailed overview of the amount of digitized objects (percentage) per Swiss canton. For each canton, there is additionally documented how many institutions have been considered for these calculations [table is based on Beer *et al.* (2)]

Canton abbreviation	Canton	Number of institutions	Amount of digitized objects (%)
AG	Aargau	4	0.15
AI	Appenzell Innerrhoden	NA	NA
AR	Appenzell Ausserrhoden	1	0
BL	Basel-Landschaft	1	0.07
BS	Baselstadt	5	0.11
BE	Bern	4	0.28
FR	Freiburg	1	0.32
GE	Genève	2	0.15
GL	Glarus	1	0.12
GR	Graubünden	1	0
JU	Jura	1	0
LU	Luzern	2	0.67
NE	Neuchâtel	4	0.20
NW	Nidwalden	NA	NA
OW	Obwalden	NA	NA
SH	Schaffhausen	1	0.11
SZ	Schwyz	1	0
SO	Solothurn	2	0.65
SG	St. Gallen	1	0.43
TI	Ticino	2	0.52
TG	Thurgau	1	0.74
UR	Uri	1	0
VS	Valais	3	0.74
VD	Vaud	4	0.27
ZG	Zug	NA	NA
ZH	Zürich	13	0.07

#### Data access and exchange

In terms of access to the collection data, all institutions want an ‘open access’ policy for their data, but report that data sovereignty should stay under the control of the individual institutions.

Most collections are involved in international communities, even though the smaller ones limit their efforts to providing data to info fauna, the Swiss National Data and Information Centre for Swiss fauna (http://www.cscf.ch/) or are only involved in specific projects.

#### Urgent problems and needs

While the scope of the problems and needs varies with the collection size, several issues prevailed across institutions of all sizes. Software issues, sustainable maintenance of the CMS, data curation issues and data sharing between different IT systems and software solutions are of major concern. There is a general call for homogenization and standardization of current practices in order to make sharing and integrating data from other collections easier. Smaller institutions in particular want to see their data used and not forgotten.

### Future vision for an SVNHC

Concerning the vision for the SVNHC, nearly all interviewed representatives demand a national solution, with the possibility of connecting and publishing to international solutions and aggregators. The SVNHC should be based on an open-source solution because this offers the possibility to tweak and adapt the system as necessary.

Most of the representatives want the future CMS to adopt a decentralized solution, with the possibility of sharing data. The need for a centralized ‘backup system’ was mentioned frequently, and interviewees suggested that such a system should be operated by a central, neutral organization which would make the individual data visible to a national and international audience.

A few main opinions emerged concerning the governance of the SVNHC. It was suggested that either a neutral organization, such as SCNAT, or a newly formed body should take responsibility for hosting and maintaining the infrastructure or that the task be given to an existing big player like InfoSpecies or GBIF, which has the reputation, expertise and resources to carry the responsibility for such a task.

Regarding financing, the unequivocal opinion was that the federal government should support the SVNHC. Collections would be willing to contribute financially, depending on the distribution key applied.

Some interviewees stressed that SwissCollNet should not reinvent the wheel but build on already existing components and network structures. On the international level, the collaboration with the network of the Consortium of European Taxonomic Facilities (https://cetaf.org/) or a large aggregator like GBIF.org could be expanded.

Representatives from smaller institutions who cannot afford to build their own independent CMS and transfer their data to national and international data aggregators pointed out that their data should be visible and that they are therefore looking forward to the SVNHC solution.

In some interviews, there was a demand for a clear statement from SwissCollNet explaining the goals of the SVNHC and its target groups.

### Existing building blocks and potential use for an SVNHC

There is already a range of national and international organizations, initiatives and infrastructure in the domain of natural history data compilation. Some of the existing structures could be extended for the realization of an SVNHC and are described in more detail below.

#### National organizations and initiatives

A comprehensive overview of national organizations and initiatives is presented on the SwissCollNet webpage (https://swisscollnet.scnat.ch/de/id/QnMZL).

InfoSpecies (https://www.infospecies.ch/de/) is the Swiss Information Centre for species, an umbrella organization of the National Data and Information Centres (Swissbryophytes, SwissLichens, SwissFungi, Info Flora, info fauna—karch, info fauna—SZKF, bats CCO|KOF and the Swiss ornithological institute). InfoSpecies coordinates and ensures the data flow between these data centres, coordination agencies, the federal government, cantons, research institutes, natural science collections and other stakeholders in species promotion. InfoSpecies shares its data with GBIF and is responsible for decisions regarding the maintenance and development of the VDC run by the Swiss Federal Institute for Forest, Snow and Landscape Research WSL.

The Swiss node of GBIF has been adopted by the Federal Office for the Environment (FOEN) in order to coordinate data quality and the homogeneity of Swiss biodiversity data shared at the national and international levels. It is run by info fauna, the Swiss National Data and Information Centre for Swiss fauna (http://www.cscf.ch/). The Swiss GBIF node currently shares ∼14 million data points from observational, monitoring, collection and DNA data. Data from GBIF publishers outside Switzerland that refers to Swiss territory can be repatriated (registration by InfoSpecies). The node is permanently financed by FOEN and BAFU.

The VDC allows users to query species’ data in raw data quality and metadata. It builds on the cooperation with the species data from the Swiss National Data and Information Centres and GBIF Switzerland, as well as content from the Nature and Landscape Data Centre. The VDC has established itself as an interface between governmental geodata and the species data in the nature conservation work of cantonal agencies. Access is limited to authorized users. Occurrences can be displayed in a map view and species lists can be generated as needed.

**Figure 2.**
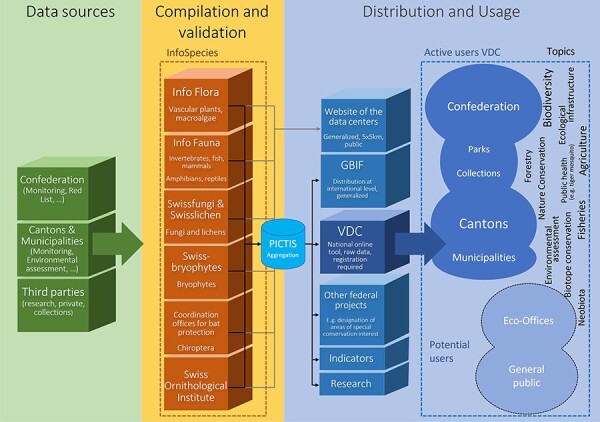
The picture shows the data flow from the data sources to the aggregation in PICTIS and the publication through the VDC to different categories of users. (Figure courtesy of Lukas Wotruba, WSL).

In order to harmonize and serve the data of the National Data and Information Centres to the VDC and GBIF, info fauna started the Oracle-based system Plateforme Informatique de Collecte, de Transfert et d’enrichissement des données Info Species (PICTIS) in 2014. The National Data Centres on Species Information collect and validate species data, which are standardized and harmonized by GBIF Switzerland. GBIF Switzerland coordinates the transfer of the data to the international Open Data Infrastructure of GBIF.org and VDC. The PICTIS database is utilized for this task (Figure 2). The FOEN uses data validated by InfoSpecies and provides them via Geographic Information System layers to the cantons and municipalities, as well as other actors like nature parks (through the VDC). The usage rights are defined by the data centres and the FOEN, as defined in the InfoSpecies guidelines (https://www.infospecies.ch/de/daten/datennutzung.html and https://www.infospecies.ch/de/assets/content/documents/2019_Deontologie_InfoSpecies_D.pdf).

#### International initiatives

##### Global Biodiversity Information Facility

GBIF was established in 1999 and is an international aggregator of biodiversity data. Today, GBIF is an international network and data infrastructure, coordinated through a secretariat in Copenhagen. It is based on common standards and open-source software (https://www.gbif.org/what-is-gbif). GBIF is organized around national nodes that implement the GBIF strategic plan and work programme, share biodiversity data under common standards and promote its use at the national level. Switzerland is a long-term member of GBIF, initially as an observer for several years and since 2016 as an official member, based on the positive decision of the Federal Council. This decision secures the sustainable funding of the Swiss GBIF node, which is run by info fauna in Neuchâtel.

As of April 2023, GBIF contained nearly 2.3 billion records (https://www.gbif.org/). The majority of the datasets shared through GBIF.org are observational records, published using the Darwin Core (https://dwc.tdwg.org/) Archive format (DwC-A). GBIF contains biological data and data from fossil collections [e.g. Paleobiology Database (https://paleobiodb.org/#/)], but no records of geological or mineral objects.

One of the big achievements of GBIF is the ‘GBIF Backbone Taxonomy’ with >7.4 million entries as of April 2023 (3). The taxonomy aims to cover all existing species names (synonyms, different languages).

GBIF offers the possibility to create a ‘Hosted Portal’ (https://dev.gbif.org/hosted-portals.html), which can be a portal website or even a virtual exhibition for a country or institution [as an example, see the development of a national portal in Norway (https://livingnorway.no/), introduction website of the ‘Living Norway Ecological Data Network’ (https://hp-living-norway.gbif-staging.org/), Hosted Portal on GBIF (https://www.gbif.no/index.html) and Norwegian node on GBIF].

On an international level, GBIF is the *de facto* standard for aggregating and disseminating biodiversity data.

##### Distributed System of Scientific Collections

DiSSCo (https://www.dissco.eu/) was initiated by European institutions to create a multinational research infrastructure aiming to provide access to over 1.5 billion natural history objects. DiSSCo is currently in the project phase of ‘DiSSCo Prepare’, running from 2020 to 2023. The goal of this phase is to improve the implementation readiness level for the construction of the research infrastructure and to deliver a DiSSCo construction master plan. According to the current road map, DiSSCo should become operational in 2025. It is planned that DiSSCo will contain both biological and geological data (plants, animals, fossils, minerals and rocks). Whereas GBIF is based on a data model that puts the physical location of an observation or a specimen in a collection at the centre of attention, DiSSCo is based on the logic of a ‘digital specimen’ (DS) model, which can be summarized as a digital surrogate for physical objects (https://dissco.tech/2020/03/31/what-is-a-digital-specimen/) with all its facets. The DS contains data about the physical specimen and links to other data/related artefacts (typically, entries in other databases). However, both DiSSCo and GBIF are currently moving to link their data with other sources to create an ‘extended specimen model’. This paradigm shift has certainly to be taken into account as the SVNHC is considered. DiSSCo is a research infrastructure that will obtain data from various providers. Whatever solution Switzerland chooses for data aggregation, it must be able to make the data available to DiSSCo (and similar platforms). Exactly how this will be done cannot be determined today because DiSSCo is still in a planning phase.

##### Geoscience Collections Access Service

The Geoscience Collections Access Service (GeoCASe) is a data network and web portal designed to make collections of minerals, rocks, meteorites and fossils held in museums and research institutions universally available online in order to foster scientific research and collaboration internationally. In its current form, it is a small aggregator with only seven institutions and 1.6 million records. Software is available on GitHub (4). In the field of earth sciences, aggregators are far less developed than in the field of biodiversity data. GeoCASe is a promising initiative that could be an alternative to Switzerland building its own aggregator for earth science data.

#### Possible software for aggregators

The following list names existing software that could possibly play a role in an SVNHC. CMS software is used at the individual institution level to manage the collection in all its detail. Aggregators stand above the individual collections and unify data from multiple collections to facilitate accessibility and increase visibility.

##### Atlas of Living Australia

The Atlas of Living Australia (ALA) is a free and open-source IT infrastructure for the aggregation and delivery of biodiversity data. The software is used not only in Australia (https://www.ala.org.au) but also in other countries like Sweden (https://biodiversitydata.se/), France (https://openobs.mnhn.fr/), Austria (https://biodiversityatlas.at) or Brazil (https://www.sibbr.gov.br/). In order to use the software in Switzerland, it would have to be adapted and installed. In addition, the various CMS in the institutions would have to be enabled to be able to publish using the ALA framework.

##### Specify

As of version 7, the CMS Specify (https://www.specifysoftware.org/)can manage several collections on one installation. In addition, Specify currently builds community functionality (Specify Network: https://www.specifysoftware.org/specify-network/) in the sense of a ‘biodiversity information architecture’ so that multiple collections can, for example, work with the same taxonomy and give each other access to specimen entries. Specify 7 is based on current web technologies, using the Python-based Django framework behind an Apache web server (https://www.specifysoftware.org/architecture-and-components/).

##### PICTIS

PICTIS, mentioned earlier, is the central data hub operated by info fauna for biodiversity data both from collections and observations. PICTIS is the backbone of the GBIF Switzerland node with a data platform for raw data with interfaces and the possibility to publish on different other aggregators and platforms. It currently contains ∼20 million data points. PICTIS is thus a good example of an existing aggregator on the national level. Data from biological collections can be transferred to PICTIS regardless of whether the specimen originates in Switzerland or abroad.

While PICTIS offers an automated upload and validation/versioning of Swiss occurrence records, there is no automated solution for the collection of data, and the current publication process from the collections to GBIF Switzerland is perceived as slow. From a node perspective, standardized datasets and institutionalized data exchange are prerequisites for increased automation and update procedures. If GBIF is to play a stronger role in the future, data exchange needs to be reinforced. The publication process must be improved, or the institutions must be able to publish on GBIF themselves through the use of a specific Integrated Publishing Toolkit. Info fauna has started to address these problems on a large scale and plans further developments.

### Requirements for an SVNHC depending on the perspectives of different stakeholders

The requirements for an SVNHC were sharpened through the interviews and combined with the requirements formulated at the beginning of the study, a concrete view of what is needed to aggregate collection data was formed.

Currently, the collections and museums struggle with digitizing their collections and bringing their CMS up to date. The following requirements for the SVNHC were formulated:

- Give better access to collection data for researchers. Today, researchers have to contact each collection individually when looking for a specimen of a certain species.
- Give better visibility to the collection data. This is generally welcomed, as long as the provenance of the data is clear. Small collections fear for the visibility of their collections in vast aggregators like GBIF.
- Functionalities of the SVNHC like virtual exhibitions or a Swiss portal website would put the collection into a larger context. This could be especially attractive for small collections.
- The requirements differ depending on the type of collection. Both biological and geological collections have CMS implemented, but the biological collections are generally more advanced with the development of CMSs. National and international infrastructures exist for the aggregation of biodiversity data. For earth science collections, standard large CMSs are still in development. For geology, Swisstopo plays a central role as it is responsible for geographic names, while further aggregation to international portals has either not occurred or occurred only to a limited extent. This is due to limited resources as well as smaller collections compared to biological ones. Furthermore, the earth science communities are generally less aware of such opportunities for interconnectedness, with GeoCASe being an exception.

Researchers need to have reliable data quality (following the FAIR principles) and access to metadata should be directly possible and easy. The aggregated data of the DS should be linked to other data like genomic data, literature excerpts or others and integrated into a bigger international research infrastructure. Research projects do not necessarily stop at the national borders. Data from Swiss collections must be put in context with data from other countries.

For public administration at the federal, cantonal and municipal levels, the collection of data is complementary to biodiversity observational data, and they can provide information on species distribution from a historical perspective.

Hobbyists in the general public are involved in collecting observational data of, e.g., Swiss flora or fauna. If data collected by hobbyists were made publicly available, it would be a way to both value their effort and provide a valuable source of information for hobby naturalists.

Environmental Consulting Offices need to comply with federal environmental and nature protection laws. Therefore, they need to have access to environmental data sets. At the moment, they do not have direct access to the VDC. Obtaining easy access to the data in the VDC would greatly facilitate their work.

The observational data producers from the different data centres think that it is important to have a central aggregation of collection specimen data (including metadata, data flow and ethical framework) within a common system, but do not seem to have special requirements regarding the SVNHC.

### Different scenarios and recommendations

With the building blocks available and the different use cases outlined, we propose several scenarios for building an SVNHC, evaluate them and assess the costs and risks.

#### Focus on data model and vocabularies

A common internal logic must be established and followed for a meaningful aggregation of collection data. The data must be based on the same or at least similar data models, use the same controlled vocabularies and ideally be based on the same logic of a persistent identifier. This first scenario proposes that the focus should rely on the data model and vocabularies with the collections establishing a joint coordination committee to address these issues and make decisions that are as binding as possible. The focus of this committee should be on ensuring that the Swiss collections and their data can be connected to large aggregators such as GBIF or future research infrastructure such as DiSSCo. The development of a separate infrastructure is not at the centre of this scenario.

The targets of this scenario would be achievable during the funding period 2021–24. This scenario would lay a long-term foundation that would help collections not only build and acquire new CMS but also take individual steps to feed their data into international aggregators (or do that with the help of info fauna). The coordination committee could continue to exist after the end of the funding period and there would be no significant fixed costs beyond 2024, except for the occasional participation of the collections in this committee.

This scenario could serve well as a sub-scenario for the following more comprehensive scenarios.

#### Building an aggregator from scratch

This scenario includes the development of a technical infrastructure and the development of a new organizational framework with sustainable funding. Biological and geological (and possibly anthropological) collection data would be united in a completely new infrastructure. One central portal would be accessible through an API and/or an end-user interface. The national aggregator would publish to international portals like GBIF or GeoCASe. The aggregator could be based on the existing software stack from ‘ALA’. The ALA software for the aggregation and delivery of biodiversity data is open source and would have to be adapted to the Swiss context. It would at least give a head start compared to a development from scratch.

A sub-scenario could be to build several aggregators for biosciences collection data and geosciences collection data (geological, mineral, fossil and meteorite specimen data) as the different types of collections are too disparate. If both kinds of data were needed for a scientific project, they could be brought together through common data analysis tools and technologies as long as common core variables (e.g. geolocation) were available.

The effort and costs of building and operating a new aggregator would be enormous, both in absolute terms and compared to the benefits it would generate over already existing infrastructure. Furthermore, such an endeavour could not be realized within the SwissCollNet funding period (2021–24). At the moment, there is no outstanding advantage in building a new infrastructure and organizational structure, as they would largely replicate existing initiatives such as InfoSpecies, GBIF.ch and VDC.

#### Collections publish directly to aggregators

In this scenario, no national aggregator would be built. The collections would be published directly to existing international aggregators like GBIF and GeoCASe. The collections would have to be supported to enable their CMS to directly publish to such aggregators. The individual collections would be responsible for the data quality and the metadata models used. Each collection would be responsible for the entire chain, from data entry, quality control, linking to controlled vocabularies, handling updates, to publication on one or more international aggregators. Publishing to the VDC would still need the involvement of InfoSpecies as an organization to connect to PICTIS and the Swiss GBIF node.

Without coordination among the collections, no common agreement on data quality may occur, different taxonomic backbones may be used and geographical indications may be disguised. To reduce these shortcomings, a standardization committee with people from the collections would have to ensure coordination among the institutions.

In general, this could be a viable approach for large collections or collections that combine their data with observational data or indications to literature. It could also be a viable option for collections connected to a larger multi-tenant CMS. Medium-sized and smaller collections with scarce resources would have problems maintaining the publication workflow to international aggregators in a sustainable way. They would especially need support and appropriate tools. The focus of the 2021–24 funding period would thus be to provide collections with tools and workflow support for publication on aggregators.

If there is no national node, the collections will have to do much more coordination among themselves, which will also generate further costs. The solution without a national aggregator is hardly more cost-effective than a solution with a central node and smaller collections could be left out due to a lack of sufficient resources to support such an endeavour. The success of this solution with exclusively horizontal coordination is questionable.

#### Support and develop existing infrastructure

SwissCollNet could fund the development and adaptation of existing infrastructure with the goal of covering the specific requirements of the different collection data stakeholders.

The national data aggregation could be based on the existing PICTIS data aggregator, which can publish data to various international platforms. For earth sciences and geological collections, additional functionalities would have to be added to PICTIS. Aggregated specimen data should then be published in international initiatives such as GBIF.org, GeoCASe and, later, DiSSCo. A very central requirement is that the publication processes are robust, automated and done in near-time for new data and updates. GBIF.org even offers the possibility of building hosted portals (https://dev.gbif.org/hosted-portals.html) for special uses. This might be an option for the SVNHC online portal.

On a national level, the data could be made available through the map server of InfoSpecies and/or the GBIF Switzerland website.

Creating a Swiss national aggregator by relying on the existing organizational and technical structures of InfoSpecies/info fauna, PICTIS and the Swiss GBIF node would both be cost-effective and reduce risk during the implementation phase. This is because the expansion of PICTIS, especially to include geodata, could be planned and implemented iteratively and incrementally. The new Swiss national aggregator for natural history collection data could profit from the investments and learnings from PICTIS.

In this scenario, the expansion of existing infrastructure should be accompanied by establishing a road map for the project planning. Such a road map would have to describe the functionalities that would be added over several stages. Each of these stages can then be calculated individually. The number of new functionalities to be created can be adapted to the available budget. The financial risks can thus be well controlled and can be kept much lower with an incremental implementation than with a completely new solution.

#### Build an ‘enriched dataset’ linkage hub

In this scenario, the solution to build on existing infrastructure would be expanded by enriching or linking to additional resources. The natural history collection data would be associated with observational data (VDC), literature (Plazi) and/or Swiss BOL/DNA data. GBIF already has a link to the VDC and SwissBOL. Thus, only the link to Plazi would need to be built.

Rather than being a self-standing scenario, this one is understood as more of an add-on that should be kept in mind for future development. Linked and enriched datasets are becoming important in several disciplines and should therefore also become a part of natural history collection datasets in the future.

## Conclusions

In our report, we recommended focusing on data models and vocabularies, plus supporting and developing the existing infrastructure in collaboration with InfoSpecies, applying the following principles:

- Coordinate vocabularies and data models among the collections.
- Focus on the development of existing infrastructure, invest in their reliability, process automation and capacity to link data between platforms To coordinate further development, experts from Swiss natural history museums have to be included in the board of InfoSpecies.
- Add functionalities where needed: add the possibility for geological data and specimen data from outside Switzerland.
- Focus on the investments and do not dilute them by funding a heterogeneous landscape of too many different portals and initiatives.
- Avoid introducing artificial heterogeneity in datasets, work on common core data sets and a common taxonomic backbone. Invest in data normalization and quality improvement.
  - ‘Catalogue of Life’ as a taxonomic backbone in GBIF (see https://www.catalogueoflife.org/).
  - Populate collection information in the ‘GBIF Registry of Scientific Collections’ (https://www.gbif.org/grscicoll) global.
- Create a Swiss portal website on GBIF which contains not only data published through PICTIS but also from large collections that might want to publish directly from their CMS Invest in a good-looking, attractive UI/UX with functionalities like virtual exhibitions. This will be an important public showcase.
- Create a sustainable business model to finance the ongoing costs, as well as to have a reliable organization with the necessary scientific and technical skills.

As a long-term goal, the natural history collections should be associated with observational data, literature and DNA data in an ‘enriched dataset’ linkage hub.

## Data Availability

The data underlying this article cannot be shared publicly due to the privacy of individuals and institutions that participated in the study. The data will be shared on reasonable request to the corresponding author with permission of surveyed third parties.
